# Profiles of mathematical deficits in children with dyslexia

**DOI:** 10.1038/s41539-024-00217-x

**Published:** 2024-02-15

**Authors:** B. Pedemonte, C. W. Pereira, V. Borghesani, M. Ebbert, I. E. Allen, P. Pinheiro-Chagas, J. De Leon, Z. Miller, B. L. Tee, M. L. Gorno-Tempini

**Affiliations:** 1 Memory and Aging Center, Department of Neurology, University of California, San Francisco, CA USA; 2 Dyslexia Center, University of California, San Francisco, CA USA; 3 Faculty of Psychology and Educational Sciences, Université de Genève, Genève, CH Switzerland; 4 Department of Epidemiology and Biostatistics, University of California, San Francisco, CA USA

**Keywords:** Education, Human behaviour

## Abstract

Despite a high rate of concurrent mathematical difficulties among children with dyslexia, we still have limited information regarding the prevalence and severity of mathematical deficits in this population. To address this gap, we developed a comprehensive battery of cognitive tests, known as the UCSF Mathematical Cognition Battery (MCB), with the aim of identifying deficits in four distinct mathematical domains: number processing, arithmetical procedures, arithmetic facts retrieval, and geometrical abilities. The mathematical abilities of a cohort of 75 children referred to the UCSF Dyslexia Center with a diagnosis of dyslexia, along with 18 typically developing controls aged 7 to 16, were initially evaluated using a behavioral neurology approach. A team of professional clinicians classified the 75 children with dyslexia into five groups, based on parents’ and teachers’ reported symptoms and clinical history. These groups included children with no mathematical deficits and children with mathematical deficits in number processing, arithmetical procedures, arithmetic facts retrieval, or geometrical abilities. Subsequently, the children underwent evaluation using the MCB to determine concordance with the clinicians’ impressions. Additionally, neuropsychological and cognitive standardized tests were administered. Our study reveals that within a cohort of children with dyslexia, 66% exhibit mathematical deficits, and among those with mathematical deficits, there is heterogeneity in the nature of these deficits. If these findings are confirmed in larger samples, they can potentially pave the way for new diagnostic approaches, consistent subtype classification, and, ultimately personalized interventions.

## Introduction

Dyslexia is a neurodevelopmental disorder characterized by difficulties with accurate or fluent word recognition, poor decoding, and poor spelling abilities, despite normal intelligence, schooling, and language exposure. This disorder affects ~5–10% of the global population and is primarily associated with reading impairment. However, increasing evidence suggests that neurodevelopmental disorders rarely occur in isolation. The rate of overlap between dyslexia and other learning disorders is extremely high, ranging from 20% to 70%. This suggests that individuals with dyslexia may also experience challenges in other cognitive domains. Specifically, children with dyslexia often exhibit lower mathematical achievement and encounter specific difficulties in various mathematical domains^1^. Around 40% of children with reading difficulties are reported to have difficulties in learning math^2–4^. Prevalence estimates for children with dyscalculia who also have dyslexia vary widely, from 17%^5^ to as high as 64%^6,7^.

Despite the evident coexistence of dyslexia and mathematical deficits, the specific profiles and underlying cognitive mechanisms that characterize the relation between linguistic and mathematical aspects of cognition are still a matter of debate. Mathematical difficulties observed in children with dyslexia are generally not specific to number processing^2–4^. Difficulties in retrieving arithmetic facts from long-term memory^8^ are common in dyslexia^9^. Impairments in specific cognitive domains (such as verbal working memory, visuospatial working memory, and lexical naming speed) could also explain the co-occurrence of deficits in mathematical cognition and reading challenges^4,10,11^.

Understanding the nature of mathematical deficits in children with dyslexia is essential for designing appropriate interventions and elucidating the complex relationship between reading and mathematical abilities. To address this knowledge gap, we designed and tested a new battery of tests, the UCSF Mathematical Cognition Battery (MCB), specifically designed to comprehensively assess mathematical deficits in a cohort of dyslexic children. The development of the MCB is rooted in the extensive body of cognitive and neuroscientific research. Here, we provide a comprehensive description of this research and elucidate its influence in shaping the selection of the four mathematical domains evaluated by the MCB: 1. number processing; 2. arithmetical procedures; 3. arithmetic facts retrieval; and 4. geometrical abilities.

Research in neuroscience^12^ provides evidence supporting a potential classification of mathematical impairments. It reveals that a selective deficit in numerical processing, which is typical in dyscalculia, is just one among several possible deficits. Specifically, difficulties in arithmetic fact retrieval, calculation abilities^13–16^, and visuospatial skills^17^ have been associated with distinct brain regions.

Moreover, multiple research groups^2,14,18–23^ provide converging evidence that emphasizes the fundamental role of four cognitive and neural domains in acquiring mathematical skills. These domains encompass number processing; arithmetical procedures; arithmetic facts retrieval; and geometrical abilities.

These four domains, which affect mathematical cognition and differ in their cognitive and neural correlates, can be differentially impacted by neurological processes and environmental factors. Consequently, they likely contribute to the classification of mathematical deficits.

Number processing skill refers to the ability to manipulate, generate, and interpret numbers. The development of the number concept requires an innate basic *number sense*^24^ or *number module*^2^ and the acquisition of its exact numerical and linguistic representations. Converging evidence indicates that humans are born with the ability to represent numbers as continuous quantities along a mentally organized number line^25–28^. Additionally, the foundation of the number concept has been associated with two nonverbal systems: subitizing, which involves quickly recognizing the cardinality of small sets of objects^29^, and the approximate number system (ANS), which enables estimation of numerical magnitude for larger sets of objects^18^. These systems are present from infancy^27,30–33^ and are shared with various animal species^31,34^.

While there is ongoing debate regarding whether subitizing abilities are linked to counting skills and other non-numerical capacities such as attention and working memory^35–39^, the ANS is considered a fundamental component of numerical cognition development^27,29,40,41^. Numerous studies have shown that ANS acuity predicts later mathematical achievement^42,43^, and it is frequently impaired in children with mathematics learning disorders^12,29^.

The brain network involved in number processing primarily encompasses the lateral parietal lobe but also extends to the inferior frontal gyri, insula, and subcortical structures^44^. With such an extensive network of brain regions, problems in mathematical reasoning can stem from various underlying computations, leading to different behavioral phenotypes.

Deficits in number processing, which characterize developmental dyscalculia, manifest through multiple symptoms in learning mathematics, including difficulties in manipulating and transcribing numbers^45^. A lack of understanding of the concept of numerosity hinders the normal development of number representation^13^, as well as number production and comprehension^14,20,21^. Children with deficits in number processing exhibit impairments in transcribing numbers across different formats (e.g., from pictures or words to Arabic numerals) and in comparing and ordering quantities^46,47^. Regarding counting, process deficits are primarily observed in counting backward, counting by twos or threes, and completing sequences^48^.

Therefore, clinical assessment of deficits in number processing should include subitizing, non-symbolic ANS (comparison between two arrays of dots), symbolic ANS (comparison between two numbers written in digit form), and principles of counting, comparisons, ordering, and transcribing between number digits and number words.

Arithmetical procedures (or calculation) skill refers to the ability to perform mental and written addition, subtraction, multiplication, or division strategies, typically without relying on counting. This skill is crucial for providing accurate mathematical solutions and plays a significant role in speeding up the problem-solving process. The activation of appropriate numerical representations is essential for executing calculations correctly^49,50^. However, deficits in arithmetical procedures may arise even when numerical skills remain intact^51–53^. These deficits may manifest as isolated difficulties in solving arithmetic problems^13,15,54^.

The brain network involved in calculation processes seems to encompass a complex system of interconnected fronto-parietal cortical and subcortical regions^55–57^.

Finger counting is often observed in children experiencing difficulties with arithmetic procedures^58^^,59^, possibly as a means to reduce the load on working memory during calculation processing^46^ or due to reliance on immature calculation strategies. Deficits in arithmetical procedures can also arise when children struggle to acquire efficient calculation strategies^16,60^, which are known to follow specific developmental trajectories^61^. For instance, a child might have difficulty transitioning from relying on external aids, such as fingers, to employing more advanced mental strategies like counting on (e.g., solving 2 + 7 = 9 by recognizing the larger addend as 7 and then counting on from eight to nine^62,63^).

When assessing calculation arithmetical procedures skills clinically, it is important to cover mental calculations, particularly addition and subtraction, as well as written calculations involving all four operations.

Arithmetic facts are simple mathematical operations that children are expected to memorize and store in long-term memory^52,64,65^. Memorizing these facts is crucial for performing quick calculations, which form the foundation of mathematical competence. Without knowledge of multiplication tables, for example, children cannot effectively learn written multiplication and division. Difficulties in mastering arithmetic facts can sometimes manifest as isolated challenges in mathematics^13,15,54^.

The retrieval and calculation of arithmetic facts involve overlapping yet distinct brain areas^66,67^. While calculations have been linked to the previously mentioned fronto-parietal network, the learning and recall of arithmetic facts appear to rely on the hippocampal, para-hippocampal, and retrosplenial structures, with the angular gyrus playing a critical role^56,68^.

Children who struggle with arithmetic facts often face difficulties in memorizing basic addition and subtraction facts (e.g., 2 + 2 = 4, 3–1 = 2) or recalling multiplication tables (e.g., the tables of 6, 7, and 8). However, if their number processing and arithmetical procedures skills remain intact, they may be able to reconstruct operation results using counting and mental strategies.

When conducting clinical assessments of arithmetic facts retrieval, it is crucial to evaluate an individual’s knowledge of these facts. The testing should include appropriate assessments of multiplication tables, taking into account the individual’s age and educational level.

Difficulties in mathematics can still be observed in children who have intact numerical skills, calculation abilities, and memory functions for recalling arithmetic facts. In such cases, deficits often lie in the non-linguistic aspects of mathematical information^69^, including visuo-perceptual, visuospatial, and visuo-constructional skills. These nonverbal abilities have been shown to predict mathematical performance, particularly in geometry acquisition^70,71^.

Geometrical knowledge is believed to be based on two core knowledge systems: an innate ability to quantitatively process visual properties (e.g., length, angle, and shape) and a later-developed ability to process specific geometrical concepts (e.g., parallel vs. perpendicular lines)^72^. Visuo-perceptual, visuospatial, and visuo-constructional abilities have been localized in the occipito-temporal^73,74^, temporo-parietal^75^, and parietal^76^ cortices, respectively, with a right hemisphere lateralization.

Deficits in geometrical abilities can manifest as visuo-perceptual difficulties in processing symbols, arranging numbers for written calculations, interpreting graphs and figures, and recognizing salient visual features of objects^13^. Symptoms may also be present in processing visuospatial information, such as spatial reasoning about orientations, directions, and distances, as well as visuo-constructional information necessary for transforming three-dimensional objects^77–79^.

When clinically assessing geometrical abilities, tasks should include (1) processing distances and directions to assess visuospatial ability, (2) matching shapes presented in different orientations or decomposed to demonstrate visuo-perceptual ability, and (3) mental rotations of 2D and 3D objects to assess visuo-constructional functions^80^. It is important to note that stimuli in 3D and 2D are processed differently in the brain^81^; for example, 3D stimuli impose a higher load on the perceptual system but are memorized better than 2D stimuli^82^.

Drawing from research in mathematical cognition and neuroscience, which supports the potential classification of four distinct mathematical deficits, we have developed and evaluated a new battery of tests called the UCSF MCB. The MCB is specifically designed to comprehensively assess the four previously mentioned mathematical domains: 1. number processing; 2. arithmetical procedures; 3. arithmetic facts retrieval; and 4. geometrical abilities. In this article, we provide a detailed description of the battery and present the results obtained from a large cohort of children (*n* = 75) referred to the UCSF Dyslexia Center with a dyslexia diagnosis, as well as 18 typically developing control children. Our focus is on classifying mathematical cognitive deficits in children with dyslexia.

## Results

### Performance in MCB for clinically defined groups

There were no group differences noted in the demographic characteristics, such as sex, handedness, and age at testing. As expected and defined, there was a significant group difference in diagnoses. However, when the controls were removed from the analysis, the significant group difference in diagnoses was no longer present (*p* = 0.318).

No significant differences were found in the distribution of mathematical deficits across grades (*p* = 0.83). However, it should be noted that this analysis may be underpowered due to the relatively low number of cases in some groups.

The UCSF-MCB confirmed the clinicians’ impression for the 18 typically developing control children and the 25 children with dyslexia but no challenges in mathematics (referred to as the Dysl_notM group in Table 1). The Dysl_notM group (*n* = 25, one-third of the dyslexia sample) and the typically developing group (referred to as the TD group in Table 1) differed significantly only in the Approximate Number System (ANS) for digits. The TD group performed significantly better than all other subgroups on the ANS for digits. It is possible that the Dysl_notM group performed worse than the TD group in this subtest because 20% of the Dysl_notM group had ADHD, and attentional deficits may affect the ANS score. Additionally, performance on the subitizing subtest revealed a main effect of group (*p* = 0.031), but post hoc analysis indicated a specific group effect only for TD compared to all other groups combined (TD mode = 6 dots; all other groups combined mode = 5; *p* = 0.016).

**Table 1:** Demographics and performance in the mathematical cognition battery (MCB)

Performance in the mathematical cognition battery									
	Controls		Deficits in mathematics						
Subtests of the DSB	TD (*n* = 18)	Dysl_notM (*n* = 25)	Number (*n* = 10)	Arithmetical procedures (*n* = 15)	Arithmetic facts retrieval (*n* = 15)	Geometrical abilities (*n* = 7)	Omnibus Sig (F,p)	*Adj model (F,p)	Omega-Sq (MR, group)
Demographics									
Sex (M:F)	11:7	19:6	4:6	9:6	12:3	4:3	0.3	n/a	n/a
Handness (R:L)	16:2	23:2	9:1	14:1	14:1	7:0	0.963	n/a	n/a
Other diagnoses (dyslexia: dyslexia and suspected ADHD)	0:0	19:6	6:5	9:6	7:8	6:1	0.288	n/a	n/a
Age at math testing (y)	10.45 (1.63)	11:69 (2.01)	11.68 (2.47)	11.73 (2.49)	12.07 (1.95)	11.97 (1.74)	0.263	n/a	n/a
Age difference (math-neuropsych)	0.01 (0.03)	0.39 (0.87)	0.38 (0.73)	0.30 (0.64)	0.26 (0.55)	1.20 (1.43)	0.118	n/a	n/a
Numbers									
Writing numbers in digit (performance in %)	95.56 (7.04)c	95.6 (8.2)c	60 (16.32)*	92.67 (10.32)c	96 (7.37)c	92.86 (11.1)c	23.42, <0.00001	16.05, <0.00001	(0.065, 0.437)
Spelling number in letters (performance in %)	97.78 (4.27)c	93.6 (9.52)c	67 (24.9)*	89.33 (13.35)c	98.67 (3.52)c	98.57 (3.78)c	12.37, <0.00001	10.52, <0.00001	(0.023, 0.340)
Sequences (performance in %)	96.30 (9.13)c,d	85.33 (23.23)c,d	30 (29.2)a,b,e,f	51.11 (27.07)a,b,e,f	85.56 (10.67)c,d	85.71 (11.5)c,d	20.37, <0.00001	16.38, <0.00001	(0.107, 0.334)
Ordering (performance in %)	94 (13.4)c,d	87.69 (18.33)c,d	47.33 (19.8)*	70.04 (19.38)a,b,c	79.2 (15.9)c	74 (18.82)c	11.39, <0.00001	6.41, <0.00001	(−0.014, 0.223)
ANS digit (performance in %)	95 (6)*	82 (12)a,c	69 (19)a,b	75 (13)a	81 (8)a	75 (10)a	7.73, <0.00001	4.82, 0.0004	(0.012, 0.142)
ANS dots (performance in %)	81 (11)f	72 (12)	68 (14)	69 (11)	71 (12)	64 (10)a	2.86, <0.02 n.s.	2.90, 0.015, n.s.	(−0.003, 0.111)
Arithmetical procedures									
Additions (performance in %)	98.33 (2.42)c	98.55 (2.78)c,d	86.13 (9.25)*	92.92 (8.6)b,c,e	98.67 (2.22)c,d	96.96 (4.07)c	11.05, <0.00001	9.75, <0.00001	(0.155, 0.105)
Subtractions (performance in %)	97.36 (4.04)c,d	96.2 (4.91)c,d	65.13 (20.09)*	80.5 (15.55)*	93.67 (8.95)c,d	95.36 (5.08)c,d	18.13, <0.00001	12.30, <0.00001	(0.057, 0.275)
Written calculation (performance in %)	88.19 (13.7)c,d	77.5 (22.46)c,d	31.88 (19.64)a,b,e,f	44.58 (27.53)a,b,e,f	68.33 (20.92)c,d	83.04 (12.87)c,d	14.93, <0.00001	9.73, <0.00001	(0.012, 0.273)
Time additions (time in seconds)	53.5 (25.54)c,d	80.08 (36.31)c,d	149.3 (59.93)a,b,f	151.47 (84.84)a,b,e,f	95.8 (35.63)d	65.29 (25.39)c,d	10.21, <0.00001	9.84, <0.00001	(0.093, 0.200)
Time subtractions (time in seconds)	66.78 (32.58)c,d,e	112.48 (66.85)d	183.1 (64.4)a	192.4 (99.6)a,b,f	142.06 (50.55)a	100.57 (41.93)d	8.42, <0.00001	5.27, 0.0002	(0.026, 0.134)
Time written calculation (time in seconds)	112 (61.46)e	210.88 (101.17)	171.1 (94.77)	216.13 (189.47)	300.8 (147.6)a	232.57 (101.71)	4.17, 0.002	3.49, 0.004 *n.s*.	(0.111, 0.092)
Arithmetic facts retrieval									
Multiplications (performance in %)	89.65 (25.1)c	85.48 (27.54)c	35.24 (34.16)*	66.90 (25.55)c	82.30 (9.07)c	96.77 (5.78)c	8.99, <0.00001	15.45, <0.00001	(0.246, 0.214)
Time multiplications (time in seconds)	43.71 (30.14)d,e	71.35 (33.41)e	57.88 (27.56)e	110.29 (69.26)a	142.47 (73.95)a,b,c	77.71 (46.65)	7.78, <0.00001	5.02, 0.0003	(−0.013, 0.268)
Geometrical abilities									
Geometrical test (performance in %)	82.69 (8.06)c,d,f	81.11 (9.03)c,d,f	61.60 (10.9)a,b,e	66.75 (15.24)a,b,e	82.28 (8.04)c,d,f	63.60 (5.5)a,b,e	12.75, <0.00001	16.11, <0.00001	(0.240, 0.269)
2D models (performance in %)	86.11 (11.25)	85.5 (13.82)	71.25 (17.72)	74.17 (20.84)	84.17 (15.28)	71.43 (13.91)	2.86, 0.02 *n.s*.	4.36, 0.0009	(0.166, −0.028)
3D models (performance in %)	88.89 (14)	95.33 (7.63)c,f	76.67 (23.83)b	84.44 (13.31)	84.44 (13.31)	76.19 (16.26)b	3.98, 0.0028	4.95, 0.0003	(0.067, 0.090)

A table showing the subtests that were used to distinguish the groups of mathematical deficit. *diff from everyone; a = diff from typically developing (TD) children; b = diff from children with dyslexia not affecting mathematical abilities (Dysl_notM); c = diff from the group with deficits in number; d = diff from the group with deficit in arithmetical procedures; e = diff from the group with deficit in arithmetic fact retrieval; f = diff from group with deficit in geometrical abilities. Adj Model is corrected for nonverbal (Matrix Reasoning) reasoning scores. Effect sizes are provided based on the more conservative adjusted model (Matrix Reasoning, group). All *p* values were Bonferroni-corrected for multiple comparisons (*p* < 0.003). All post hoc pairwise comparison *p* values were Bonferroni-corrected for multiple comparisons (*p* < 0.01).

The Dysl_notM group performed well on the MCB subtests evaluating number processing, arithmetical procedures, arithmetic facts retrieval, and geometrical abilities. Some of these participants obtained low scores only on subtests assessing teaching exposure (e.g., 8% obtained a low score in the “equivalent fractions” subtest, and 18% in the “percentage” subtest) or more complex mathematical skills (e.g., 30% obtained a low score in the “simplifying expressions” subtest, and 36% obtained a low score in the “solving equations” subtest).

**Fig. 1:**
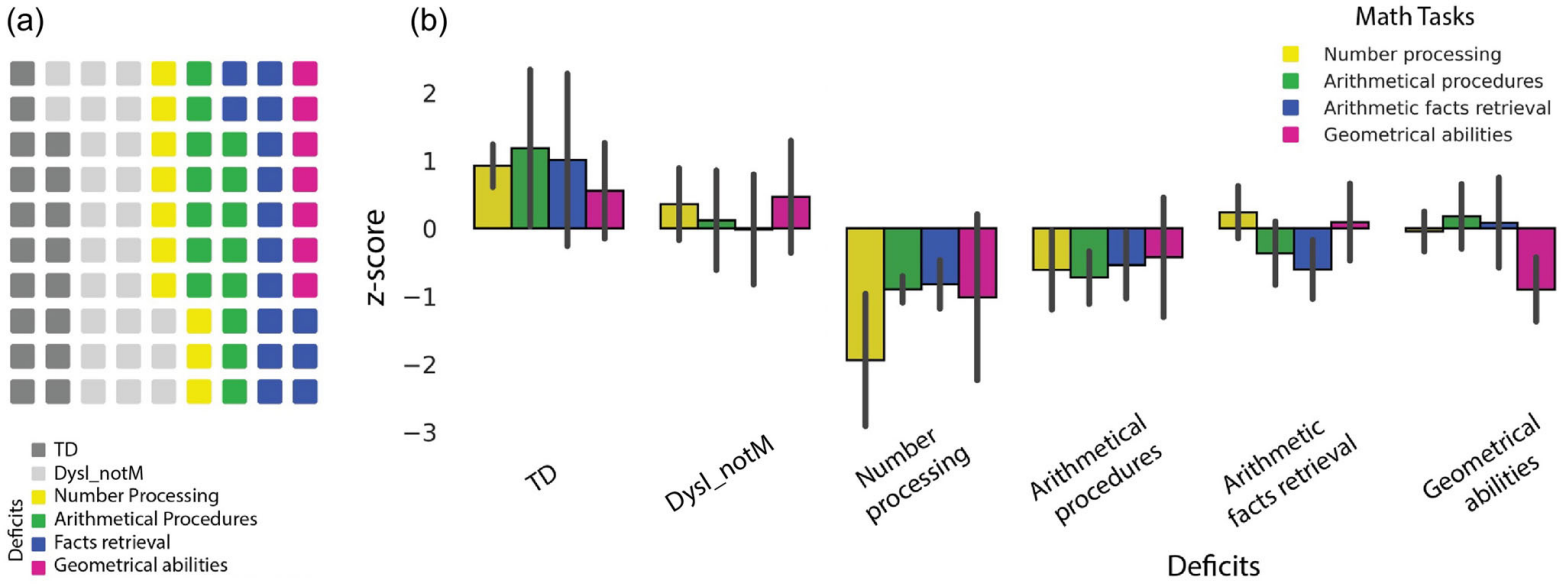
Group performance in MCB. **a** The distribution of each group across our sample (*n* = 90): typical developing controls (TD, *n* = 18), dyslexia and no challenges in mathematics (Dysl_notM; *n* = 25), deficits in number processing (*n* = 10), deficits in arithmetical procedures (*n* = 15), deficits in arithmetic facts (*n* = 15), or deficits in geometry (*n* = 7). **b** Standard score performance on tasks weighing on number processing (yellow), arithmetical procedures (green), arithmetic fact retrieval (blue), and geometrical abilities (purple), for the six groups identified: typically developing (TD) children, children with dyslexia not affecting mathematical abilities (Dysl_notM), children with deficits in number processing, arithmetical procedures, arithmetic facts retrieval, and geometrical abilities. Errors bars represent standard errors of the mean. Details on the different subtests (including examples of incorrect responses) are in Supplementary Table 1.

The other 50 participants from our dyslexic cohort (50/75, 66.6%), classified by the clinicians’ team as having deficits in mathematics, performed poorly on specific subtests of the MCB corresponding to their specific deficit (refer to Fig. 1b). Significant group differences were found in all subtests of the MCB math battery (refer to Table 1), except for the 2D shape reconstruction test (refer to Supplementary Table 1, # 26).

Children with deficits in number processing (*n* = 10, 13.3% of dyslexia sample) performed significantly lower than all other groups on number subtests and obtained low scores in most of the MCB’s subtests. They showed a pattern of weakness in at least three of the number subtests (shown in Supplementary Table 1, #1–8) or a severe deficit in comparing, ordering, or transcoding numbers from one representation to another with lexical or syntactical mistakes (see Supplementary Table 2 for inclusion and exclusion criteria). Lexical mistakes refer to instances when digits are incorrectly chosen, but the order of magnitude is correct, e.g., 250 instead of 215. Syntactical mistakes refer to instances when digits are correctly chosen, but the order of magnitude is incorrect, e.g., 20,053 instead of 2053. Children with deficits in number processing performed significantly lower than all other groups in the addition and subtraction subtests of the calculations section and the multiplication subtest of the arithmetic facts section, which suggests that deficits in number processing represent more fundamental deficits in learning mathematics. They also performed worse than TD and Dysl_notM subgroups on the ANS for digits.

Children with deficits in arithmetical procedures (*n* = 15, 20% of dyslexia sample) showed a pattern of weakness in at least one of the three calculation subtests (Supplementary Table 1: #9. Mental additions, #10. Mental subtractions, #13. Written calculation) or were accurate but slow at calculations (see Supplementary Table 2 for inclusion and exclusion criteria. Notably, they performed significantly differently from all other groups on the subtraction subtest: the completion time is the highest of all groups, and the performance is lower than the other groups, except for those with deficits in number processing. The group of children showing deficits in arithmetical procedures also had low scores on the sequencing subtest (Supplementary Table 1, #3). They correctly identified the pattern in each sequence but made mistakes in calculating the answer. Surprisingly, the arithmetical procedures group exhibited comparable performance to children with number processing deficits. Notably, they did not demonstrate strong performance in the number processing subtests. However, their scores in these particular subtests were still relatively higher than those of the number processing group. In general, the group showing deficits in arithmetical procedures performed better on most subtests than the group showing deficits in number processing but worse than the other subgroups.

Children with deficits in arithmetic facts retrieval (*n* = 15, 20% of dyslexia sample) obtained low scores or accurate but slow performance on multiplication and division problems (Supplementary Table 1: #11. Mental multiplication and #13. Written Calculation). They demonstrated a specific deficit in the time to complete the multiplication table (see Supplementary Table 2 for inclusion and exclusion criteria). They also took the longest to complete the written calculation subtest, which also includes multiplication and division.

Children with deficits in geometry (*n* = 7, 9.3% of dyslexia sample) performed lower than TD group, Dysl_notM group, and the group with deficits in arithmetic facts retrieval on the computerized geometrical test (Supplementary Table 1, #27). They showed a pattern of weakness in the geometry subtests (Supplementary Table 1: #25. 2D shape reconstruction, #26. From 2D shapes to 3D models) performing significantly lower on the 3D models than that Dysl_notM group (see Supplementary Table 2 for inclusion and exclusion criteria). However, they performed not significantly different from the group with deficits in number processing on both computerized geometrical subtest and the 3D models subtest. The group with deficits in geometrical abilities was the only group to perform worse than TD on the ANS for dots (*p* = 0.032). In this subtest, numbers are represented through dots, which might be confusing for children who have trouble perceiving forms and identifying shapes. Visual impairments were excluded.

Three participants (4% of dyslexia sample) were excluded from further analyses because their deficits overlapped in arithmetic procedures and fact retrieval, and a clear subgroup could not be determined.

In summary, among the 75 participants referred with a diagnosis of dyslexia, 50 had trouble in mathematics (i.e., two-thirds of dyslexia sample). The clinician’s assessment was confirmed for all but three (*n* = 47) of the children in the dyslexia group. Only 13.3% of dyslexia sample showed impairment in number processing (*n* = 10). Most children (40% of the dyslexia sample) had impairments in arithmetical procedures (*n* = 15) or arithmetic facts retrieval (*n* = 15). Only 9.3% of dyslexia sample showed impairments in geometry (*n* = 7). See Fig. 1a for more details.

### Performance in the WJ-IV calculation test

In the WJ-IV Calculation test, around 30% of children with no apparent difficulties in mathematics (8 out of 25 children) obtained a result below the 16 percentiles outside the average range (any score that falls one standard deviation below the mean indicates below-average performance). However, not one of these children obtained a low score in prior mathematical assessments using the WJ-IV (Broad Math, Applied Problems, Fluency), Wechsler Individual Achievement Test (WIAT; Math, Numerical, Problem Solving, Fluency), Kaufman Test of Educational Achievement (KTEA; Math Concepts and Applications, Computation, Fluency, Problem Solving), or the Feifer Assessment of Mathematics^83^. Furthermore, their previous and current history did not indicate any symptoms of mathematical difficulty.

On the other hand, ~25% of the children with dyslexia and mathematics difficulties (12 out of 47) obtained a score on the WJ-IV Calculation test at or greater than the 25th percentile (average performance); of these children, none had deficits in number processing, five had deficits on arithmetic facts retrieval, three on arithmetical procedures, and four on geometrical abilities.

### Group performance in neuropsychological and academic assessments

Despite the small number of children in each group with impairment in mathematics, we nevertheless present results in neuropsychological and academic tests. Among the groups with dyslexia, statistically significant differences were also noted in the performance of WJ-IV Calculations (WJ Calc), nonverbal reasoning (Matrix Reasoning), and judgment of line orientation (JLO) tests (Table 2). Specifically, the group with Dysl_notM had higher scores on the WJ Calc and Matrix Reasoning subtests than the groups showing deficits in number processing and arithmetical procedures. There were also group differences on JLO, but the Dysl_notM and the group with a deficit in arithmetic facts retrieval performed better than the group with a deficit in number processing.

**Table 2:** Neuropsychological performance in percentile score based on ANOVA

	Dysl_notM	*#*	Numb	*#*	Arith Proc	*#*	Arith Facts	*#*	Geom	*#*	Sign. (*p*; omega-sq)
	Ave(std)		Ave(std)		Ave(std)		Ave(std)		Ave(std)		
General cognitive and academic											
WJ-IV oral vocabulary	60.8 (25.7)	24	35.8 (38.1)	7	47.6 (21.3)	14	52.7 (21.7)	15	61.7 (25.9)	7	0.16
Receptive vocabulary -ROWPVT	72 (25.2)	23	57.4 (34.8)	7	64.7 (23.2)	14	77.8 (16.1)	14	85.5 (16.2)	7	0.14
WASI matrix reasoning	72 (27.3)c,d	23	24.4 (30.4)b	7	34.7 (23.3)b	14	55.1 (22.8)	15	56.1 (23.4)	7	**<0.001; 0.275**
WJ-IV calculations	37.4 (26.7)c,d	24	3.6 (3.4)b	7	13.2 (13.6)b	14	21.5 (19.5)	15	28.7 (10.4)	7	**0.001; 0.218**
Reading											
(TOWRE-2) sight words	24.6 (24.4)	24	4.16 (6.7)	7	15.2 (13.5)	14	25.5 (23.5)	15	18.9 (25.4)	7	0.16
(TOWRE-2) pseudowords	19.1 (18.8)	24	3.4 (4.5)	7	15.5 (10.6)	14	19.8 (22.8)	15	14.9 (19.8)	7	0.3
Processing speed											
WISC-IVi symbol search	55.7 (27.8)	22	21.8 (12.1)	6	29.5 (28.0)	13	42.5 (26.3)	15	44.6 (17.5)	7	0.016
WISC-IVi coding	25.7 (27.9)	22	11.4 (14.1)	7	14.9 (14.5)	13	23.3 (13.6)	15	29.1 (28.0)	7	0.344
CCT 1 (timed number seq)	41.5 (32.3)	23	34.5 (34.0)	7	30.7 (29.2)	12	38.3 (26.9)	15	32.4 (27.6)	7	0.865
Visuospatial processing											
Judgment of line orientation	50.9 (34.2)c	23	6.4 (15.3)b,e	8	22.9 (30.1)	12	46.6 (22.2)c	14	36.7 (24.6)	7	**0.002; 0.195**
Beery VMI	40.1 (25.5)	23	15.1 (10.6)	8	22 (26.3)	13	32.3 (25.1)	15	12.0 (6.1)	7	0.015
Memory											
Short-term verbal - WISC-IVi- digits forward	44.8 (30.6)	23	20.9 (16.2)	7	39.4 (25.3)	14	38.9 (26.5)	15	31.0 (27.2)	7	0.322
Long-term verbal - CVLT LDFR	53.7 (31.7)	22	33.2 (23.8)	6	41.7 (31.2)	14	58.9 (33.6)	14	67.7 (19.5)	7	0.18
Short-term visual - WISC-IVi- blocks forward	43.8 (28.7)	19	31.3 (27.9)	7	38.5 (31.3)	11	37.4 (27.4)	14	40.1 (20.8)	7	0.886
Long-term visual - Rey-Osterrieth figure 3’ delay	39.2 (34.81)	22	12.4 (20.8)	7	14.0 (16.9)	14	47.8 (36.7)	15	13.0 (18.3)	7	0.005
Executive functions											
Flanker	39.5 (27.2)	16	24.6 (32.1)	5	35.5 (30.9)	11	50.2 (28.7)	12	28.8 (26.1)	4	0.456
Verbal working memory- WISC-LVi- digits backward	39 (23.1)	23	10.6 (7.5)	7	31.7 (28.5)	14	42.1 (21.1)	15	35.6 (30.6)	7	0.06
Visual working memory - WISC-LVi- blocks backward	49.4 (32.0)	18	22.6 (15.6)	7	29.2 (23.8)	11	30.4 (24.9)	14	49.0 (31.7)	7	0.082
CCT 2 (timed number seq/switch)	37.9 (20.3)	23	12 (12.3)	7	16.5 (18.8)	12	26.0 (20.9)	15	25.7 (17.4)	7	0.008
DKEFS design fluency-filled	56.1 (22.4)	19	49 (32.9)	6	47.6 (24.2)	11	66.3 (25.6)	15	43.0 (43.6)	4	0.331

# = number of participants; b = diff from Dysl_notM, no deficits in mathematics; c = diff from the group with deficits in number; d = diff from the group with a deficit in arithmetical procedures; e = diff from the group with deficit in arithmetic fact retrieval. The statistical significant differences are indicated in bold. All omnibus *p* values Bonferroni-corrected for multiple comparisons (*p* < 0.003), all post hoc pairwise comparison *p* values Bonferroni-corrected for multiple comparisons (*p* < 0.0125).

Children with deficits in number processing obtained the lowest scores in all neuropsychological assessments except for the CCT 1 (timed ordinal number sequencing), the Beery VMI, and the DKEFS Design Fluency-filled. Post-hoc analyses revealed they performed significantly worse than the Dysl_notM group on WJ Calc, Matrix Reasoning, and JLO tests (Table 2; all *p*’s < 0.05 Bonferroni-corrected). They also had significantly lower scores on JLO than the arithmetic fact retrieval group (*p* < 0.05 Bonferroni-corrected). Most deficits appeared in tests that require visual reasoning and judgments, but verbal knowledge was spared.

Children with deficits in arithmetical procedures obtained lower scores than the Dysl_notM group on WJ Calc and Matrix Reasoning.

Children with deficits in arithmetic facts retrieval did not perform worse than other groups in any of the neuropsychological tests. They had the highest group average of the dyslexia sample on untimed line degree matching (JLO) and performed significantly better than the children with deficits in number processing. The children with deficits in arithmetic facts retrieval also had the highest scores on geometry subtests of the MCB among the groups with math difficulties.

Children with deficits in geometry obtained good scores in general cognitive and academic tests, as well as in processing speed tests. Even though not significantly different, their scores on the visuomotor test (Beery VMI) were the lowest. They also had a low score in long-term visual recall (Rey-Osterrieth Figure 3-minute Delay). Notably, both these tests require drawing.

## Discussion

There is a notable lack of comprehensive studies that have systematically examined various components of mathematical skills in cohorts of children with concurrent learning difficulties. Despite the significant prevalence of mathematical deficits among children with dyslexia (average 40%^4^), our understanding of the frequency and severity of these deficits in this population remains limited.

To address this gap, we have developed and evaluated the UCSF MCB, a set of subtests specifically designed to classify mathematical cognitive deficits in children with dyslexia. The MCB was developed at UCSF Dyslexia Center (UCSF-DC) and tailored for children ranging from 2nd to 8th grade. It spans number processing, arithmetical procedures, arithmetic facts retrieval, and geometrical abilities, allowing for personalized educational practices and interventions based on individual profiles. In this study, we present the initial results of a large cohort of children referred with a diagnosis of dyslexia (*n* = 75) who were carefully evaluated by a group of expert clinicians. The MCB confirmed the clinicians’ mathematical impressions, demonstrating its potential as a promising assessment tool that can be scaled to larger cohorts of children. In the following discussion, we explore the clinical and educational implications of our findings and their relationship to current neurocognitive theories of mathematical deficits.

The main result of our study is that the MCB can effectively identify mathematical impairments in children with dyslexia. Among our cohort, 50 out of the 75 participants referred with a diagnosis of dyslexia at the UCSF-DC were found to have deficits in mathematics, which were rarely identified in their previous evaluations. This suggests that many cases of mathematical difficulties are currently going undetected. The commonly administered assessments such as WJ (Calculation Test, Broad Math, Applied Problems, Fluency), WIAT (Math, Numerical, Problem Solving, Fluency), KTEA (Math Concepts and Applications, Computation, Fluency, Problem Solving), and FAM may not capture difficulties in mathematics, particularly if those difficulties are not severe or related to number deficits.

Moreover, the currently available mathematical assessments do not effectively distinguish between deficits in number processing, arithmetical procedures, arithmetic facts retrieval, and geometrical abilities. Specifically, geometry is under-assessed in psychoeducational evaluations and should be included in the assessment of mathematical cognition. Identifying difficulties in geometry early on is crucial because it is possible to detect these difficulties even before formal instruction begins (which typically occurs in the 10th grade in the US). Children possess an innate capacity for geometrical intuition from an early age, as they are sensitive to shapes in both 2D and 3D and can recognize relationships between shapes and forms^72,84–86^. Difficulties in geometrical abilities can also impact a student’s ability to use mathematical symbols correctly (e.g., using “x” for multiplication and “+” for addition) and interpret graphs and charts, which are essential for tests in sciences and math, standardized placement tests, and job skills^87^.

A significant finding of our study highlights the potential of the MCB to classify mathematical deficits in children with dyslexia (refer to Supplementary Table 2: preliminary diagnostic decision guide). By assessing the participants’ performance and completion time on the MCB, dyslexic children with mathematical impairments can be categorized into one of four groups representing specific mathematical deficits: number processing, arithmetical procedures, arithmetic facts retrieval, and geometrical abilities. Two groups (the number processing group and the geometrical abilities group) demonstrated lower performance in the specific subtests associated with their respective subgroup (refer to Fig. 1). Conversely, the arithmetical procedures group and the arithmetic facts group did not show significantly poorer performance in their corresponding subtests. It is worth noting that the number processing group displayed inferior performance across the subtests related to arithmetical procedures and arithmetic facts retrieval. However, the arithmetic facts retrieval group exhibited the lowest scores in the subtests designed to identify challenges in arithmetic facts retrieval. Similarly, the arithmetical procedures group demonstrated the lowest scores in the subtests designed to pinpoint difficulties in calculation.

We will briefly explore potential criteria to describe the main characteristics of each group and the likely neurocognitive correlates.

Deficits in number processing are characterized by difficulties in understanding and manipulating numerical quantities. Children with deficits in number processing struggle with comprehending the magnitude of numbers, estimating quantities, and grasping numerical relationships. They may face challenges in counting, recognizing numbers, comparing numbers, and ordering them.

In the UCSF MCB, deficits in number processing were observed in at least three of the following subtests: translating numbers between different representations (digits, words, pictorial), comparing and ordering numbers, and completing number sequences. Sometimes deficits may also be observed in subitizing, and more frequently in the Approximate Number System (ANS), both in symbolic (digits) and non-symbolic (dots) formats. We found no significant difference in the group of children with a number processing deficit compared to the other groups in the subitizing test. However, these children often struggled to provide an accurate estimation of the number of dots when the quantity exceeded five. They would often state that there were 30, 40, 50, or 100 dots, even when the maximum number of dots on the screen was nine. In the MCB, children with a deficit in number processing performed worse than the control group and the other subgroups on the ANS for digits. While all groups tended to perform better on digits than dots, the children with a deficit in number processing only showed a slight improvement in digits compared to dots (a difference of one point), whereas the other groups showed greater differences (minimum difference: 6, maximum difference: 14). This further highlights the pervasive difficulties experienced by children with a deficit in number processing, regardless of the presentation format.

Numbers form the foundation of calculations, logical reasoning, and problem-solving; therefore, a deficit in numbers inevitably compromises many aspects of mathematical thinking^88^. It is not surprising that deficits in number processing have a detrimental impact on all other mathematical skills, as indicated by the low scores observed in this subgroup across the entire battery. These children exhibited impairments in calculation skills and geometrical abilities. Furthermore, the positive relationship between numerical skills and visuospatial skills^36,89–92^ may help explain the low scores obtained by these participants in the geometrical subtest. In these tests, no significant difference was observed between children with a deficit in number processing and children with a deficit in geometrical abilities in the geometrical subtests.

Additionally, children with a deficit in number processing demonstrated difficulties in various tests that require visual reasoning and judgment but showed preserved verbal knowledge. This suggests that a cognitive mechanism involving higher-order nonverbal reasoning and attention may underlie the difficulties observed in this subgroup.

It is plausible to consider that this subgroup represents the co-occurrence of dyslexia and dyscalculia. Dyscalculia is characterized by impairments arising from difficulties in connecting a number to its corresponding magnitude (number sense for Cohen & Dehaene^24^; number module for Butterworth^2^). Children with deficits in number processing often exhibit this difficulty through mistakes in counting, comparing and ordering numbers, and identifying patterns in numerical sequences.

In our sample, only 13.3% (10 out of 75) of the children with dyslexia were diagnosed with deficits in number processing. This indicates that only a small portion of dyslexic children who struggle in mathematics may also have dyscalculia.

Deficits in arithmetical procedures are characterized by a focal impairment when applying arithmetic procedures. While not showing deficits in numerical skills, children with deficits in arithmetical procedures struggle with understanding, applying, and reproducing mental and/or written calculations.

In the UCSF MCB, these children performed significantly differently from the other subgroups, except for the number subgroup, both in terms of accuracy and speed in calculations. Errors include treating subtraction as a commutative operation (e.g., 5–3 ≠ 3–5) and inverting the order of digits (e.g., 30−11 = 21 because 3–1 = 2, and 1–0 = 1). Slow processing was often due to the adoption of ineffective counting strategies, often supported by fingers or drawing. However, this group did not demonstrate a significant difference in performance compared to the number subgroup, making it challenging to precisely characterize their mathematical deficits. It is possible that they share similarities with the number subgroup, displaying mild difficulties. Alternatively, it is more likely that their difficulties stem from cognitive mechanisms unrelated to the mathematical domain, indicating that other factors might contribute to their challenges.

Unexpectedly, children with a deficit in arithmetical procedures performed poorly on some of the geometry subtests. A common denominator for this pattern of difficulties could be working memory, as previously suggested^11,93,94^. However, in our study, this group did not show group differences in working memory measures. Although the group sizes are relatively small, the standard deviations overlap with the means, and the sample includes individuals with co-occurring dyslexia, so it is possible that the results are concealed within this sample, indicating a potential sampling effect. Another possibility is that this group may have some visuospatial difficulties that make it challenging for them to keep numbers aligned during arithmetical procedures, leading to errors of alignment^53^. However, we do not have evidence to support this possibility in our data (only a few errors are due to misalignment of numbers in columns). Nonetheless, more than 50% of the children with deficits in arithmetical procedures (8 out of 15) incorrectly calculated the difference between two numbers, subtracting the smaller digit from the larger without respecting the order of subtraction with regard to the whole number (e.g., in 90 – 47, they subtract 0 from 7 and 4 from 9). This error could be associated with deficits in the spatial representation of quantitative information^95^ because the “direction” of the operation is incorrectly interpreted. They may also rigidly apply one rule, “subtract the smaller number from the larger,” at the expense of other rules, which may suggest difficulties in ranking competing choices.

It is also possible that when performing written calculations, we are constantly dividing our attention. If someone has proficient calculation skills and knowledge, they may divide their attention between the motor program of writing and the estimation of the correct result in order to check their work simultaneously. However, a child who has not mastered calculations may instead divide their attention between the motor program of writing and searching their mind for the appropriate calculation rules/algorithms for those numbers. If this is the case, interventions for this type of deficit may focus on alternate strategies that reduce the burden of divided attention.

Deficits in arithmetic facts are characterized by impairments in arithmetic facts retrieval. Children with deficits in arithmetic facts do not show difficulties in number subtests, and they are able to perform calculations. However, they struggle to recall the results of operations that should have been learned through rote memorization, such as multiplication facts. The deficit in recalling multiplication tables is coupled with difficulties in writing multi-step multiplications and divisions.

In the UCSF MCB, children with a deficit in arithmetic facts took the longest time to complete the multiplication table subtest and the written calculation subtest. It is crucial to evaluate not only the overall performance but also the response time: slow responses are likely associated with compensatory strategies that are only partially effective. For instance, complex multiplication can be solved by relying on the lengthy mental strategy of repeated addition (e.g., 8 × 4 is broken down into 8 + 8 = 16 and then 16 + 16 = 32). Children with deficits in arithmetic facts generally performed well in addition and subtraction tasks. Subtraction and multiplication are associated with distinct neural systems for numerosity and language^96^.

Children with deficits in arithmetic facts retrieval also had the highest scores on the geometry subtests of the MCB. One potential explanation is that this group may have developed enhanced visuospatial skills either in response to or because of their language difficulties. Balancing resources across the brain may lead to increased functionality in visuospatial skills to compensate for diminished functionality in language. Although fluency in reading and arithmetic are considered distinct abilities, significant correlations have been found between them^97,98^. Due to the similarities between the initial stages of reading and mathematical fluency development, it is likely that the same brain and cognitive mechanisms are involved in both domains^99^. Since our cohort was recruited based on symptoms of dyslexia, it is difficult for us to distinguish these differences. Future studies using the MCB to evaluate the performance of children with dyslexia compared to dyscalculia could help address this question.

Deficits in geometry are characterized by impairments in geometrical abilities. Children with deficits in geometry exhibit difficulties in the nonverbal representations of mathematical information. They may struggle to process distances and directions, match shapes, recognize geometrical transformations (e.g., symmetries, rotations), and mentally reconstruct a 3D model from a 2D shape.

In the UCSF MCB, children with deficits in geometrical abilities obtained the lowest scores on the geometry subtest and performed significantly worse on the 3D model test compared to the typical development (TD) group, the group with no deficits in math, and the group with deficits in arithmetic facts. In the computerized test^72^, they made mistakes in solving problems related to geometrical transformations (symmetries, rotations, and translations), distinguishing distances, and identifying characteristics of geometrical figures.

Notably, the group with a deficit in geometrical abilities was the only one to perform worse than the TD group on the Approximate Number System (ANS) for dots. There is a positive correlation between numerical skills and visuospatial skills, so it is not surprising that these individuals struggled to connect numbers with visual objects^11,36,89,90,92^.

Recent research suggests that spatial ability predicts performance in mathematics^100^, but it remains unclear which mathematical skills are involved. Our group of children with deficits in geometry is not large enough to draw conclusions on this point, and further research is necessary to validate our findings.

The existing research examining the relationship between dyslexia and mathematical impairments is limited and still underdeveloped. Interestingly, cognitive and neuropsychological research, along with an increasing body of evidence, including neuroimaging studies, has accepted the existence of different subtypes of dyslexia. This raises the possibility of subtyping difficulties in mathematics as well.

For instance, core deficit dyscalculia has been associated with abnormalities in the left IPS^48,101,102^. Moreover, arithmetic problem-solving difficulties have been associated with aberrant responses (hyperactivity and hyper-connectivity) in a number of posterior brain areas suggesting a critical role of parietal circuits in deficits related to arithmetical procedures^103^. Conversely, the medial temporal lobe, specifically the left hippocampus, has been implicated in arithmetic facts retrieval^104,105^. Finally, posterior inferior-temporal cortex (including the fusiform gyrus) and the posterior parietal cortex have been linked with geometry problem solving^106^, but further studies might aim at disentailing the specific neural correlates associated with visuo-perceptual, visuospatial, and visuo-constructional deficits.

The emerging line of research indicates that mathematical impairments may exhibit different profiles and underlying mechanisms, similar to the subtypes observed in dyslexia.

The MCB has not yet been standardized, and the current criteria rely on clinical and qualitative evaluations. Additionally, the MCB is relatively long and includes subtests that might be redundant (Supplementary Table 1: #14: Find the missing sign in the expression, #15: Find the missing number in the expression, #16: True/False) or unnecessary in identifying deficits in mathematics (Supplementary Table 1: #6: Estimation, #18: Percentage, #22: Name Figure, #24: 2D shape reconstruction). Other subtests appear to simply differentiate typically developing children from children with dyslexia but do not provide information on the specific deficits (subitizing and ANS for digits).

We have identified four subtypes of mathematical deficits: number processing, arithmetical procedures, arithmetic facts retrieval, and geometry. This is a preliminary classification of mathematical deficits, and further distinctions may be necessary. For example, transcoding numbers and understanding the magnitude of numbers are skills associated with different neurocognitive profiles^107^, suggesting that deficits in number processing might be subdivided into two distinct groups.

Given the developmental nature of learning difficulties related to mathematics, it is possible that mathematical deficits may present differently at different ages and/or be partially compensated through other cognitive strengths, such that one may only show difficulty when math reasoning becomes more complex, and the learning environment is less scaffolded. Therefore, future work should be careful to include skill assessments at varying points of development through adulthood.

Three subjects showed a pattern of errors so diverse as to preclude labeling in a specific subtype. As for all neurocognitive continuums that can be broken down into clinically meaningful subtypes, there will likely always be cases that cannot be ascribed to one specific category^108^. However, future studies might help to elucidate the unique cognitive and neural correlates of these mixed cases.

Finally, the cohort of children we tested (*n* = 93) is relatively small and unequal in group size, preventing more advanced statistical comparisons of the distinct mathematical deficits. It would be helpful to replicate these findings in larger studies and with reliability metrics for the MCB.

Despite recognizing the limitations in our approach, we believe that the MCB provides a foundation for clinically relevant and neurocognitively informed diagnoses and models. Currently, we are still in the development stage of the battery in which we are relying on detailed clinical observation to determine relevant subtests following a behavioral neurology methodological approach. We are currently administering a second version of the MCB, based on the present results, to increase our sample size with an independent group of subjects which will allow us to conduct refined psychometric standardization and to identify cut-off scores for each group. We plan to use the MCB to assess children who only have mathematical learning differences to investigate whether the four deficits align with subtypes of dyscalculia.

## Methods

### The UCSF MCB

The UCSF MCB is a comprehensive experimental battery developed to identify and differentiate difficulties in various mathematical domains, allowing for the identification of individual mathematical strengths and weaknesses. The MCB is designed for students ranging from the 2nd to the 8th grade and consists of seven different forms tailored to each specific grade level. It encompasses a total of 19 subtests, including 4 computer-based subtests and 15 paper-based subtests, targeting number processing, arithmetical procedures, arithmetic facts retrieval, and geometrical abilities. Additionally, eight subtests are included to evaluate more complex mathematical skills such as simplifying expressions, solving equations, and geometrical problems, while also assessing the level of teaching exposure, which is crucial for diagnosing learning differences in mathematics. Examples and detailed information can be found in Supplementary Table 1.

To evaluate the ability to recognize and compare magnitudes expressed through digits, words, or arrays of dots, eight subtests were designed, consisting of five paper-based problems and three computer-based tasks adopted from previous literature^109^.

Four subtests were specifically created to assess arithmetical procedures skills, with three focusing on mental calculation skills and the fourth evaluating written calculation abilities. The addition and subtraction problems within the calculation subtests were designed to target difficulties related to arithmetic procedures, while a mental multiplication subtest and the multiplication and division problems in the written calculation subtest aimed to evaluate arithmetic facts retrieval skills. Additionally, the time taken to complete the calculation subtests was recorded to assess fluency, which can help differentiate between difficulties in arithmetic procedures and arithmetic facts retrieval.

To assess geometrical abilities, a computerized task^72^^,^^110^ and three paper-based subtests were included. These tasks involved items that varied in terms of symmetry, rotation, shape, angles, and other relevant aspects.

Furthermore, three tasks were added to evaluate mathematical abilities in older children, such as simplifying expressions, solving equations, and modeling, in order to detect instances when individuals might have compensated for basic deficits but still experience difficulties with more complex problems. Additionally, seven supplementary tasks were designed to verify adequate teaching exposure and to confirm or exclude specific deficits, such as fractions. For instance, a deficit in arithmetical procedures could be confirmed when solving word problems if the appropriate operation is identified, but the calculation is performed incorrectly.

### Participants

Participants were recruited through the UCSF-DC, a multidisciplinary research center dedicated to studying dyslexia and related neurodevelopmental cognitive disorders. At the UCSF-DC, participants who were referred due to concerns of dyslexia underwent a comprehensive research evaluation conducted by a team of clinicians, including neurologists, neuropsychologists, genetic counselors, speech and language pathologists, psychiatrists, and educational specialists. This team provided an overall diagnostic impression based on various factors, including clinical history (first symptoms and most severe impairments reported by parents and teachers), family history (similarities between siblings and/or parents), standard neuropsychological and academic testing, and questionnaire responses indicating clinical significance. Participants were excluded from the study if they exhibited borderline or impaired general cognitive scores, had a known history of severe perinatal events such as strokes or acquired brain injuries, or had genetic, neurological, or psychiatric disorders associated with seizures, impaired sensory processing, or aphasia. Inclusion criteria required fluency in English and an age between 7 and 16 years. The group of typically developing control participants consisted of volunteers recruited through advertisements and families expressing interest in participating in the study. Typically developing control participants had no subjective concerns regarding academic achievement, no prior diagnoses of neurodevelopmental disorders, an age range between 7 and 16 years, and fluency in English.

The final study cohort consisted of 93 children, including 18 typically developing children (7 female, mean age = 10.40 ± 1.66, 94% right-handed), 50 children diagnosed with dyslexia, and 25 children diagnosed with dyslexia and suspected attention-deficit/hyperactivity disorder (ADHD; 27 female, mean age = 11.78 ± 2.05, 92% right-handed). Detailed demographics can be found in Table 1.

Most children in the clinical group (57 out of 75, 76%) attended independent schools specifically tailored to children with learning differences, allowing their teachers to provide detailed descriptions of their academic challenges.

The guardians of the participants provided informed written consent, and the participants themselves provided assent. The study was approved by the University of California San Francisco (UCSF) Institutional Review Board and complied with the Declaration of Helsinki.

### Study procedure

**Clinical classification**. As part of the UCSF-DC diagnostic process, the team of clinicians considered each child’s clinical history, conducted teacher interviews, performed cognitive and academic evaluations, and determined whether the child exhibited difficulties in mathematical cognition, and, if so, which aspect was most affected: number processing, arithmetical procedures, arithmetic fact retrieval, or geometrical abilities. The assessment of these children’s mathematical abilities was not solely based on standardized assessments since their previous diagnoses primarily focused on cognitive and linguistic abilities. However, for 70 out of the 75 participants (93%), mathematical abilities were also evaluated using the Woodcock–Johnson IV Test of Academic Achievement, Calculation Subtest.

The clinical evaluation process began with a comprehensive assessment of the participant’s clinical history, which carried significant weight in the overall evaluation. If parents and teachers did not report any difficulties in math learning, but the participant’s performance on a math subtest was below average, the implications of the poor performance were carefully examined. Possible attributions such as ADHD, anxiety, reading difficulties, or subthreshold math deficits were considered. Clinical impressions were formed based on a convergence of positive historical evidence of mathematical learning difficulties, teacher-reported challenges in mathematics, and either notably low performance on any math subtest (falling below the 5th percentile) or below-average scores across multiple math subtests.

From the clinical assessment, 50 out of the 75 participants with a previous diagnosis of dyslexia were classified as having difficulties in some aspect of mathematics (66.6%). Among these 50 children, 10/50 (20%) were judged to have an overall impairment in mathematics, experiencing difficulties in understanding basic concepts such as numbers and magnitudes. These children were described as struggling to process numbers correctly. For example, teachers often reported that these children had trouble connecting numbers to their corresponding magnitudes, while parents recalled instances during early learning when their child struggled with counting or understanding differences in quantities of objects. The team classified these participants as having difficulties in number processing. The majority of children who struggled in mathematics (32/50, 64%) appeared to understand numbers but still faced challenges in calculation activities. Among them, 16 children experienced difficulties primarily in mental or written calculations, while the other 16 had impairments in memorizing multiplication tables and math facts. The team classified these participants as having deficits in arithmetical procedures and arithmetic fact retrieval, respectively. Finally, 8 out of the 50 children (16%) who struggled in mathematics were described as having difficulty with mathematical activities involving orientation, direction, distance, and the processing of visuospatial information (such as reading graphs and comparing similarities and differences in figures). These children did not have trouble with calculations but struggled to understand mathematical concepts when presented visually. The team classified these participants as having visuospatial difficulties, which we identified as impairments in geometrical math abilities.

In summary, based on the clinician assessment, 25/75 children were classified as not having trouble in mathematics (33.3%). Among the 50 children with math difficulties, 10/75 were classified as having impairments in number processing (13.3%), 16 in arithmetical procedures (21.3%), 16 in arithmetic fact retrieval (21.3%), and 8 in geometrical abilities (10.7%). The UCSF Dyscalculia MCB was not used for the clinical impression evaluation.

The 75 dyslexic participants and the 18 typically developing children were tested with the UCSF MCB) to investigate whether new measures of mathematical cognition could be used to identify different mathematical deficits in this cohort of children.

On average, each participant was tested for a total of 1 hour and fifteen minutes. Performance and time were recorded for each subtest. Each child was assessed with the battery tailored to the child’s grade level. Children tested during the first three months of the scholastic year (until Christmas break) were evaluated with the battery tailored to their previous grade level to help alleviate didactical confounds. In total, 12 children were tested on the 2nd grade battery form, 6 on the 3rd grade form, 16 on the 4th grade form, 23 on the 5th grade form, 12 on the 6th grade form, 8 on the 7th grade form, and 16 on the 8th grade form.

### Neuropsychological and academic assessment

Neuropsychological and academic testing were administered or supervised by a licensed neuropsychologist. The tests covered screening of nonverbal reasoning (WASI - Wechsler Abbreviated Scale of Intelligence, Matrix Reasoning^111^), vocabulary (ROWPVT - Receptive One-Word Picture Vocabulary Test^112^ and WJ-IV: Woodcock–Johnson, Oral Vocabulary^113^), processing speed (WISC-IV - Wechsler Intelligence Scale for Children, Symbol Search, Coding^111^, and CCT 1 - Color Trails Test^114^), attention and working memory (Digit and Spatial Spans), verbal and visual recall (CVLT - California Verbal Learning Test^115^ and Rey-Osterrieth Test^116^), visuospatial and visuo-construction abilities (Judgment of Line Orientation, Rey-Osterrieth^116^, and Beery VMI - Beery Visual-Motor Integration^117^), and executive functions (DKEFS - Delis-Kaplan Executive Function System, Design Fluency, Flanker^118^, and CCT 2^113^; please refer to Table 2 for a full list of tests). Academic testing was conducted using the Woodcock–Johnson IV^113^. In addition to some of the untimed reading measures in the WJ-IV, participants also underwent administration of the Test of One-Word Reading Efficiency, version 2^119^.

### Statistical analysis

Demographic, neuropsychological, and MCB measures were compared across groups (refer to Table 1). Group differences in MCB were evaluated to potentially classify mathematical cognitive deficits in children with dyslexia.

The data were analyzed using Stata 15 (StatCorp, College Station, TX). Parametric data were analyzed using ANOVA and independent sample *t* tests, while non-parametric data were analyzed using chi-squared analyses. Tests for unequal variances were employed as appropriate. A Bonferroni correction was applied to account for multiple comparisons. All statistical analyses were reviewed and, if necessary, revised by a statistician. The statistical analysis of group performance in the UCSF MCB is presented along with a general description of the performance in clinically defined groups. The z scores were calculated based on the average of each group.

### Reporting summary

Further information on research design is available in the Nature Research Reporting Summary linked to this article.

### Supplementary information

Supplementary Material

Reporting summary

## Data Availability

The datasets generated and analyzed during the study are not publicly available because not all research on our project has been completed. The data are available from the corresponding author upon completion of the study, with protected health information excluded per HIPAA requirements, and pending any other university obligations or requirements regarding data sharing.

## Code availability

No undisclosed code was used in this study.
