# Supplementary material for: Profiles of mathematical deficits in children with dyslexia
Source: NPJ Sci Learn. 2024 Feb 15;9:7. doi: 10.1038/s41539-024-00217-x (PMC10869821; doi:10.1038/s41539-024-00217-x)
Supplement: Supplementary file 1 — Supplementary Material [file 41539_2024_217_MOESM1_ESM.pdf]

Title:

Profiles of mathematical deficits in children with dyslexia

Running Head

Mathematical deficits in dyslexia

Pedemonte, B.,[1,2*], Watson, C.[1, 2], Borghesani, V.[3], Ebbert, M.[1,2], Allen, I.E.[4], Pinheiro-Chagas, P. [1,2], De Leon, J.[1,2] , Miller, Z.[1,2] , Tee B.L.[1,2], & Gorno-Tempini, M.L.[1, 2]

[1] *Memory and Aging Center, Department of Neurology, University of California San Francisco*

[2] *Dyslexia Center, University of California, San Francisco, CA*

[3] *Centre de Recherche de l'Institut Universitaire de Gériatrie de Montréal, Université de Montréal, Montréal, QC, Canada / Département de Psychologie, Université de Montréal, Montréal, QC, Canada*

[4] *Department of Epidemiology and Biostatistics, University of California, San Francisco*

Supplementary Materials

Supplementary Table 1: Description of the subtests in the MCB

Domain	Subtest (#)	Description	Example	Incorrect response example
num	1	Write numbers in digits	$Eighty - nine \longrightarrow 89$	$Eighty - nine \longrightarrow 98$
num	2	Write numbers in words	$69 \longrightarrow Sixty - nine$	$69 \longrightarrow Sixty - six$
num/ calc	3	Fill out sequences	$Fill\ out$: 10 8 _ 4 2 _	10 8 7 4 2 1
num	4	Comparing numbers	$Circle\ the\ bigger\ number$: 35 53	35 53
num	5	Ordering Numbers	$Order\ from\ least\ to\ greatest$: 100 101 110 132 92 103	92 100 103. 110 101 132
num	6	Estimation	$51 + 69 =$	$51 + 69 = 100$
num	7	Subitizing	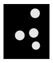	$There\ are\ 5\ dots$
num	8	Approximate number system	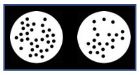	$There\ are\ more\ dots\ on\ the\ right$
calc	9	Mental additions	$Find\ the\ sum$: $19 + 5$	$19 + 5 = 23$
calc	10	Mental subtractions	$Find\ the\ difference$: $12 - 5$	$12 - 5 = 8$
fact	11	Mental Multiplications	$Find\ the\ product$: 6×9	$6 \times 9 = 56$
calc	12	Multiples of numbers	$List\ out\ the\ multiples\ of\ 4\ until\ the\ number\ 32$	$4, 8, 12, 17, 21, 25, 29, 32$
calc / fact	13	Written calculation	$33 + 26 =$ $90 - 47 =$ $16 \times 72 =$ $108 \div 9 =$	$90 - 47 = 57$
num / calc / fact / did	14	Find the missing sign in the expression	$(1 + 8)\ ?\ 5 = 45$	$? = +$
num / calc / fact /did	15	Find the missing number in the expression	$88 = ? + 80$	$88 = 18 + 80$
num / calc / fact / did	16	True/False	$Is\ it\ true\ or\ false?$ $4 \times (2 + 10) = 18$	$True$
num / did	17	Equivalent Fractions	$\frac{1}{2} = \frac{?}{6}$	$\frac{1}{2} = \frac{2}{6}$
num / did	18	Percentage	$0.5 = \frac{\quad}{100} = \_\%$	$0.5 = \frac{5}{100} = 5\,\%$
num / calc / fact / did	19	Word problems	$How\ do\ you\ share\ 24\ cupcakes\ equally\ between\ 8\ chil$	$2\ cupcakes\ for\ child$
did	20	Simplifying expressions	$9 + 8 \div (-4) - 2$	$9 + 8 \div (-4) - 2 = 2$
did	21	Solving equations	$9 - 3x = 1 + x$	$x = 5$
geometry	22	Name the figure	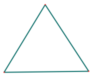	$Rectangle$
geometry / did	23	Perimeter and Area	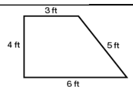	$Perimeter = 17\ ft$ $Area = 360\ ft^2$
did	24	Modeling	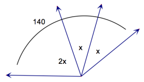	$x = 70$
geometry	25	2D shape reconstruction	$Can\ you\ make\ the\ shape\ using\ the\ two\ pieces\ in\ the\ b$ 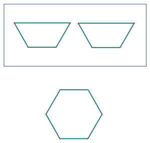	No 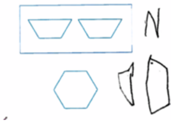
geometry	26	From 2D shape to 3D model	$Which\ 3D\ model\ does\ the\ figure\ in\ the\ box\ create?$ 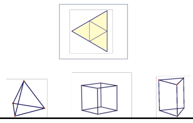	$Model\ number\ 3$
geometry	27	Geometry-Test	$Which\ figure\ does\ not\ belong\ to\ the\ set?$ 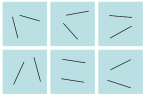	$Figure\ \#\ 6$

Supplementary Table 1: Description of the subtests in the MCB

Did = These subtests are used to eliminate or confirm didactical issues.

Num = These subtests are used to identify deficits in number processing

Calc = These subtests are used to identify deficits in arithmetical procedures

Fact = These subtests are used to identify deficits in arithmetic facts retrieval

Geometry= These subtests are used to identify deficits in geometry

Supplementary Table 2: Preliminary diagnosis decision guide

**Deficits in number processing**	
**Inclusion**: at least one of criteria 1-3 must be answered positively	
1.	A severe deficit in writing numbers in digits, e.g. performance $\leq$ 40% correct
2.	A severe deficit in ordering and sequencing numbers
3.	A systemic pattern of weakness (e.g. performance $\leq$ 70% correct) in at least three different number subtests, e.g. converting between numbers, words, digits; number comparisons; ordering; sequencing; and approximate number system.
**Exclusion**: criteria A. must be answered negatively	
A.	Didactical issues (e.g., the child has not learned a mathematical concept yet)
**Deficits in arithmetical procedures**	
**Inclusion**: at least one of criteria 1-3 must be answered positively:	
1.	A severe deficit in at least one of three calculation subtests (mental addition, mental subtraction, or written multi-step calculations)
2.	A systemic pattern of weakness (e.g. average performance < 70% correct) on calculation subtests
3.	Accurate but slow calculations (e.g. average performance above 70% correct but average time to complete each subtest is > two minutes (up to three minutes for children in the 4th grade or lower).
**Exclusion**: criteria A. and B. must be answered negatively	
A.	Didactical issues (e.g., the child has not learned a mathematical concept yet)
B.	Deficit in problems involving numbers (e.g., converting between numbers, words, digits; number comparisons; ordering; sequencing; and approximate number system)
**Deficits in arithmetic facts retrieval**	
**Inclusion**: Both criteria (1 & 2) with at least one of the conditions "a" or "b" must be answered positively:	
1a.	A deficit in recalling the multiplication table, e.g. performance $\leq$ 70% correct
1b.	Accurate but slow multiplication performance, e.g. time to complete $\geq$ two minutes
2a.	A severe deficit in performance on written multi-step calculation subtest
2b.	Written multi-step calculation subtest take more than two minutes for children in 5th grade or above and greater than four minutes for children up to 4th grade.
**Exclusion**: criteria A. and B. must be answered negatively	
A.	Didactical issues (e.g., the child has not learned a mathematical concept yet)
B.	Deficit in problems involving numbers (e.g., converting between numbers, words, digits; number comparisons; ordering; sequencing; and approximate number system)
**Deficits in geometry**	
**Inclusion**: Both criteria (1 & 2) with at least one of the conditions "a" or "b" must be answered positively:	
1.	A deficit in the computerized geometrical subtest e.g. performance < 65% correct for children up to 4th grade and < 75% for children in 5th grade or above
2a.	A systemic pattern of weakness (e.g average performance < 70% correct on the three geometrical subtests (2D model, 3D model and computerized subtest)
2b.	Average time to complete each geometrical subtest is more than two minutes.
**Exclusion**: criteria A. and B. must be answered negatively. *Deficits in Geometry may be present with other types of deficits as long as it is the predominant area of weakness.*	
A.	Didactical issues (e.g., the child has not learned a mathematical concept yet)
B.	Visual impairments (e.g., convergence problems)

Supplementary Table 2: Preliminary diagnostic decision guide

In this tables are described the preliminary inclusion and exclusion criteria for the diagnosis of deficits in mathematics based on the MCB
