# Supplementary material for: Profiles of mathematical deficits in children with dyslexia
Source: NPJ Sci Learn. 2024 Feb 15;9:7. doi: 10.1038/s41539-024-00217-x (PMC10869821; doi:10.1038/s41539-024-00217-x)
Supplement: Supplementary file 2 — Reporting summary [file 41539_2024_217_MOESM2_ESM.pdf]

nature portfolio

Corresponding author(s): Bettina Pedemonte

Last updated by author(s): Feb 23, 2023

Reporting Summary

Nature Portfolio wishes to improve the reproducibility of the work that we publish. This form provides structure for consistency and transparency in reporting. For further information on Nature Portfolio policies, see our Editorial Policies and the Editorial Policy Checklist.

Statistics

For all statistical analyses, confirm that the following items are present in the figure legend, table legend, main text, or Methods section.

n/a	Confirmed	
☐	☒	The exact sample size (*n*) for each experimental group/condition, given as a discrete number and unit of measurement
☐	☒	A statement on whether measurements were taken from distinct samples or whether the same sample was measured repeatedly
☐	☒	The statistical test(s) used AND whether they are one- or two-sided<br>*Only common tests should be described solely by name; describe more complex techniques in the Methods section.*
☐	☒	A description of all covariates tested
☐	☒	A description of any assumptions or corrections, such as tests of normality and adjustment for multiple comparisons
☐	☒	A full description of the statistical parameters including central tendency (e.g. means) or other basic estimates (e.g. regression coefficient) AND variation (e.g. standard deviation) or associated estimates of uncertainty (e.g. confidence intervals)
☐	☒	For null hypothesis testing, the test statistic (e.g. F, t, r) with confidence intervals, effect sizes, degrees of freedom and P value noted<br>*Give P values as exact values whenever suitable.*
☒	☐	For Bayesian analysis, information on the choice of priors and Markov chain Monte Carlo settings
☒	☐	For hierarchical and complex designs, identification of the appropriate level for tests and full reporting of outcomes
☐	☒	Estimates of effect sizes (e.g. Cohen's d, Pearson's r), indicating how they were calculated

Our web collection on statistics for biologists contains articles on many of the points above.

Software and code

Policy information about availability of computer code

Data collection	REDCap LTS Version 13.1.10
Data analysis	Data were analyzed using Stata 15 (StatCorp, College Station, TX). ANOVA, independent sample student's t-tests, and chi-squared analyses were used for parametric and non-parametric data respectively, using tests for unequal variances as appropriate. All pairwise and multiple comparisons used a Bonferroni correction.

For manuscripts utilizing custom algorithms or software that are central to the research but not yet described in published literature, software must be made available to editors and reviewers. We strongly encourage code deposition in a community repository (e.g. GitHub). See the Nature Portfolio guidelines for submitting code & software for further information.

Data

Policy information about availability of data

All manuscripts must include a data availability statement. This statement should provide the following information, where applicable:

- Accession codes, unique identifiers, or web links for publicly available datasets
- A description of any restrictions on data availability
- For clinical datasets or third party data, please ensure that the statement adheres to our policy

The datasets generated and analyzed during the study are not publicly available because not all research on our project have been completed. They are available from the corresponding author on reasonable request.

Human research participants

Policy information about studies involving human research participants and Sex and Gender in Research.

Reporting on sex and gender	The final study cohort included 93 children, 18 typically developing children (female = 7, mean age = 10.40 (± 1.66), 94% right-handed), 50 children with a diagnosis of dyslexia, and 25 with a diagnosis of dyslexia and suspected ADHD (female = 27, mean age = 11.78 (± 2.05), 92% right-handed).<br>The information about gender has not been collected because not meaningful in our study.
Population characteristics	See above
Recruitment	At the UCSF-DC participants were referred for a concern of dyslexia. Most children of the clinical group (57/75, 76%) attended independent schools for children with learning differences. The group of typically developing control participants were volunteers recruited through advertisement and from families who expressed interest in participating at the study.<br>Participants were excluded from the study if they showed borderline or impaired general cognitive scores, known history of severe perinatal events such as strokes, an acquired brain injury, genetic, neurological, or psychiatric disorder associated with seizures, impaired sensory processing or communication. Inclusion criteria included fluent in English, and have an age between 7 and 16 years.<br>Typically developing control participants had no subjective concerns about academic achievement, no prior diagnoses of neurodevelopmental disorders, age ranging between 7 and 16 years, and fluency in English.
Ethics oversight	UCSF Dyslexia Center (UCSF-DC), a multidisciplinary research center dedicated to the study of dyslexia and related neurodevelopmental cognitive disorders.

Note that full information on the approval of the study protocol must also be provided in the manuscript.

Field-specific reporting

Please select the one below that is the best fit for your research. If you are not sure, read the appropriate sections before making your selection.

☐ Life sciences ☒ Behavioural & social sciences ☐ Ecological, evolutionary & environmental sciences

For a reference copy of the document with all sections, see nature.com/documents/nr-reporting-summary-flat.pdf

Behavioural & social sciences study design

All studies must disclose on these points even when the disclosure is negative.

Study description	This study presents a novel battery of tests, the UCSF Dyscalculia Subtyping Battery (DSB) specifically designed to comprehensively assess four mathematical domains 1. number processing; 2. arithmetical procedures; 3. arithmetic facts retrieval; and 4. geometrical abilities. We describe the battery, and report results obtained in a large cohort of children (n=75) referred to the UCSF Dyslexia Center with a diagnosis of dyslexia and 18 typically developing control children, focusing on demonstrating the diagnostic utility and feasibility of the DSB battery. The study is mainly qualitative but we also report some quantitative preliminary results.
Research sample	The final study cohort included 93 children, 18 typically developing children (female = 7, mean age = 10.40 (± 1.66), 94% right-handed), 50 children with a diagnosis of dyslexia, and 25 with a diagnosis of dyslexia and suspected ADHD (female = 27, mean age = 11.78 (± 2.05), 92% right-handed).
Sampling strategy	Children referred at the UCSF- DC with a diagnosis of dyslexia were excluded from the study if they showed borderline or impaired general cognitive scores, known history of severe perinatal events such as strokes, an acquired brain injury, genetic, neurological, or psychiatric disorder associated with seizures, impaired sensory processing or communication. Inclusion criteria included fluent in English, and have an age between 7 and 16 years.<br>The group of typically developing control participants were volunteers recruited through advertisement and from families who expressed interest in participating at the study.<br>Typically developing control participants had no subjective concerns about academic achievement, no prior diagnoses of neurodevelopmental disorders, age ranging between 7 and 16 years, and fluency in English.
Data collection	Data were mainly collected in paper and pen.<br>Three tests of the battery are computerized. We used Matlab for two of them and java for another one.<br>Data were gathered in REDCap LTS Version 13.1.10 .<br>During the collection of data none was present beside the participant and the researcher.
Timing	September 2018 - October 2020
Data exclusions	See above
Non-participation	No participants dropped out/declined participation to the study

Randomization	Participants were not allocated in experimental groups.<br>Most children of the clinical group (57) attended independent schools for children with learning differences.

Reporting for specific materials, systems and methods

We require information from authors about some types of materials, experimental systems and methods used in many studies. Here, indicate whether each material, system or method listed is relevant to your study. If you are not sure if a list item applies to your research, read the appropriate section before selecting a response.

Materials & experimental systems

n/a	Involved in the study
☒	☐ Antibodies
☒	☐ Eukaryotic cell lines
☒	☐ Palaeontology and archaeology
☒	☐ Animals and other organisms
☐	☒ Clinical data
☒	☐ Dual use research of concern

Methods

n/a	Involved in the study
☒	☐ ChIP-seq
☒	☐ Flow cytometry
☒	☐ MRI-based neuroimaging

Clinical data

Policy information about clinical studies

All manuscripts should comply with the ICMJE guidelines for publication of clinical research and a completed CONSORT checklist must be included with all submissions.

Clinical trial registration	n/a
Study protocol	The Institutional Review Board (IRB) at UCSF, must review all research that involves human subjects performed by UCSF faculty, staff, or students or researchers at UCSF-affiliated institutions before the research can begin.
Data collection	Data were collected at UCSF, Mission Bay, 675 Nelson Rising Ln, San Francisco, CA 94158, Zoom if during covid-19, or at local schools
Outcomes	Our primary outcome measure was performance on the novel battery.
